# OMICtools: an informative directory for multi-omic data analysis

**DOI:** 10.1093/database/bau069

**Published:** 2014-07-14

**Authors:** Vincent J. Henry, Anita E. Bandrowski, Anne-Sophie Pepin, Bruno J. Gonzalez, Arnaud Desfeux

**Affiliations:** ^1^Haute-Normandie-INSERM ERI-28, Institute for Research and Innovation in Biomedicine of Rouen University, 76183 Rouen, France, ^2^Center for Research in Biological Systems, University of California, San Diego, 9500 Gilman Dr. La Jolla, CA 92093, USA and ^3^STATSARRAY, 76300 Sotteville-lès-Rouen, France

## Abstract

Recent advances in ‘omic’ technologies have created unprecedented opportunities for biological research, but current software and database resources are extremely fragmented. OMICtools is a manually curated metadatabase that provides an overview of more than 4400 web-accessible tools related to genomics, transcriptomics, proteomics and metabolomics. All tools have been classified by omic technologies (next-generation sequencing, microarray, mass spectrometry and nuclear magnetic resonance) associated with published evaluations of tool performance. Information about each tool is derived either from a diverse set of developers, the scientific literature or from spontaneous submissions. OMICtools is expected to serve as a useful didactic resource not only for bioinformaticians but also for experimental researchers and clinicians.

**Database URL:** http://omictools.com/

## Introduction

Rapid advances in next-generation sequencing (NGS), microarray, mass spectrometry (MS) and nuclear magnetic resonance (NMR) technologies have transformed biological and biomedical research over the past few years (1–4). The analysis of ‘omic’ data is a fast-evolving field, with the constant development of new statistical methods (4–8). As such, recommendations on how to analyze ‘omic’ data often change from year to year. For example, a number of new tools have been developed as part of the 1000 Genomes Project (9), but many of them have still not been published, fully vetted or subjected to peer review (10). In addition, tool details and access often change following the original publication (11), rendering it more and more challenging for research groups to stay current. There is an urgent need of organizing the bioinformatics resources (12). Among the existing efforts to solve the problem are the SEQanswers wiki (13), the NAR online Molecular Biology Database Collection (14), the Bioinformatics Links Directory (15) and the SIB bioinformatics resource portal (16). Except the SEQanswers wiki, a specific database of tools for NGS analysis, these projects do not focus on the different analysis steps of ‘omic’ applications despite the wide range of interested users. Furthermore, these resources do not provide visual guidance for biomedical researchers/life scientists with little computing experience. It is thus important to identify better ways of disseminating useful information to the scientific community.

To help remedy these deficiencies, we present OMICtools, the first open-access didactic directory that provides an overview of >4400 software tools and databases with particular attention to NGS, microarray, MS and NMR data analysis. All tools have been classified and detailed information provided. Furthermore, published evaluations of tool performance have been added to provide guidance in the choice of programs or databases. Finally, an interface has been established to allow anyone to rate a tool, ask a question and report a problem for a specific tool. By making this resource available, we aim to help experimental researchers/clinicians find appropriate tools for their needs and developers to stay up to date and to avoid redundancy. The directory can also be used by life scientists as an educational or quick-reference support.

## Catalog of bioinformatics tools

### Database construction

OMICtools (http://omictools.com/) is a metadatabase with particular attention to NGS, microarray, polymerase chain reaction (PCR), MS and NMR technologies. The directory includes >4400 tools.

**Figure 1.**
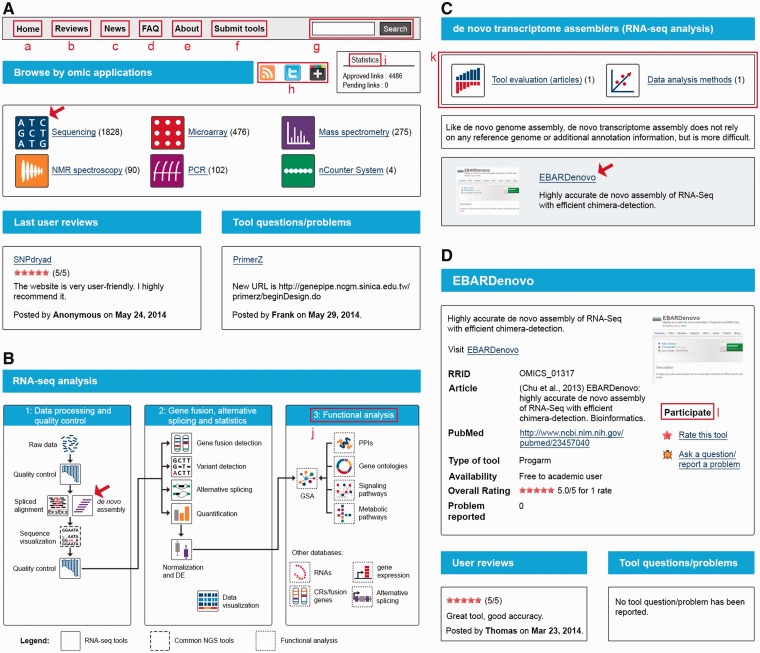
OMICtools structuration. (**A**) Classification by technologies. (**B**) Classification by analytical steps, as illustrated by RNA sequencing analysis. (**C**) List of tools for a given analytical step, as illustrated by de novo assembly (**D**) Tool description. Several features are highlighted. (a) Homepage button. (b) User reviews. (c) Latest tools added to the directory. (d) FAQ. (e) About us. (f) Link to the submission page. (g) Global site search bar. (h) Widgets that allow users to share this page with their social networks. (i) Statistics. (j) Associated databases. (k) Associated published evaluations of algorithm performance and data analysis methods. (l) Interface for interacting with the user community. Illustrated pages will be open by clicking on the icons indicated by the red arrows

Content is presented in a three-level classification format. At the first level, specific icons label the technologies (sequencing, microarray, MS, etc.; Figure 1A). At the second level, a didactic scheme labels the applications (DNA-seq, gene expression microarray, MS-based proteomics, NMR-based metabolomics, etc.). For each application, not only software tools are supplied but also associated databases. At the third level, the analytical steps specific to each application are used for classification (quality control, spliced alignment, de novo assembly, etc.; Figure 1B). For each analytical step, published evaluations of tool performance and data analysis methods are provided when they are available (Figure 1C). An exhaustive list of the different categories is available at http://omic tools.com/allcategories.html.

Analytical software and databases are linked to the directory, and clicking on each tool leads to a structured description of the tool (Figure 1C). In addition, an interface has been developed to allow anyone to report a problem, ask a question or rate a given tool, in an attempt to develop an interactive community (Figure 1D).

### Data description

**Figure 2.**
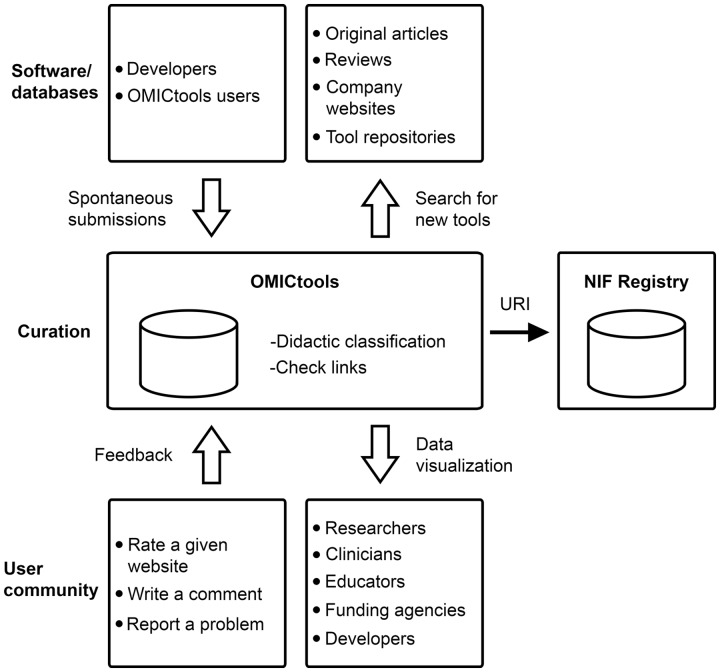
A simplified workflow of OMICtools for data dissemination and reproducibility

Analytical tools have been collected either by spontaneous submissions or by the authors from original articles, reviews, company Web sites and tool repositories (Figure 2).

Submitters do not have to register, and submission is free. Developers and users can submit software or databases to the most appropriate category. Upon request, editors can manually add tools to other categories (up to three cross-references). To improve the clarity of OMICtools, we suggest that submitters add tools with multiple applications to the common tool category. Mandatory information is kept to a minimum: tool name, tool description, Web site address and webmaster's e-mail address. However, submitters are encouraged to add more specifications: original reference, PubMed link, conditions of use (free to all users, free to academic users, license purchase required), associated biological technology, type of tools (database, link to literature, program), user interface, operating system, program language, parallel computation and licensing. Tools are reviewed by editors within two business days.

OMICtools is also continually updated by the authors to classify and include the very latest analytical tools (Figure 2). To facilitate these curation tasks, authors use an RSS feed reader, which aggregates web content such as original articles, reviews and tool repositories in one location for easy viewing. Tool specifications are then extracted and incorporate into the metadatabase. Importantly, it is manually curated with special attention to the quality of links. To ensure this, OMICtools has an automatic link checker to avoid dead links. Obsolete tools are not eliminated but transferred in the sections named ‘Deprecated tools’. A unique Research Resource Identifiers (RRIDs) have been added for each tool and transfer to the Neuroscience Information Framework (NIF) registry (see Discussion section; Figure 2).

Except where otherwise noted, content on this site is licensed under a Creative Commons Attribution-ShareAlike 4.0 International License.

### Implementation

To facilitate future development and updates of software and databases, the OMICtools database structure and software architecture has been flexibly designed. The directory is powered by a free and open-source directory script named Arfooo. The Arfooo Directory has been developed using the latest technologies [MVC architecture, Javascript/Ajax (jQuery), PHP 5] and uses UTF8 encoding. The ability to share to Twitter and Google+ is provided. An RSS feed of the latest added tools is also supplied.

## Discussion

To profitably exploit the latest ‘omic’ research techniques, it is essential to retrieve information existing in the literature. Since its launch in 2013, OMICtools has been providing a metadatabase for software and database dissemination.

OMICtools is not the only online resource providing an extensive catalog of tools (13–18). The majority of these directories focus either on software tools or databases and on a particular application (13). OMICtools differs in scope. It is the first metadatabase that combines software tools and databases and extends to all high-throughput technologies.

Many directories have used wikis after the launch of popular Wikipedia in 2001 (19). Wikis are Web sites where communities of users can collaborate online to build content and discuss progress. Wikis are extremely easy to use and edit, requiring little to no technical skill. Advantages in one context may be disadvantages in another. Contributors are free to classify tools under any kind of novel method or targeted function. However, this may cause an overrepresentation of terms and makes it difficult to find what one is looking for. Furthermore, contributors are only a small portion of the total number of people (0.02–0.03%) who access the services for information (20). Thus, many analytical tools are either missing or obsolete. OMICtools differs from wiki platforms in format and has developed new features. Firstly, while retaining the ability for spontaneous submissions, OMICtools is mainly maintained by the authors and regularly updated. Secondly, we have created a three-level classification to sort analytical tools according to their technology, application and their analytical steps. Thirdly, a schematic workflow with clickable regions is provided for each ‘omic’ application to guide newcomers. Fourthly, to provide guidance in the choice of programs, literature that evaluate algorithm performance and data analysis methods have also been added for each analysis step. This establishes a clear overview and helps non-bioinformatician readers to rapidly find the right tool. Lastly, an interface has been established to allow anyone to report a problem, write a comment or rate a given Web site, in an attempt to develop an interactive community. Feedback from our user is a crucial element in helping to prevent propagation of erroneous information, and update requests from users are dealt with as a priority.

Over the past year, Nature has published a string of articles that highlight failures in the reliability and reproducibility of published research (collected and freely available at go.nature.com/huhbyr). Starting in February, about 25 journals, including the Journal of Neuroscience and Neuron, have introduced editorial measures to address the problem by improving the consistency and quality of reporting on software tools and databases in life-sciences articles. Scientific reproducibility is dependent on many attributes of the scientific method, but one of the most fundamental is to know which materials or tools are being used. Being able to the uniquely identify these tools requires at least a unique identifier for each tool. The Resource Identification Initiative is designed to help researchers cite the key resources used to produce the scientific findings reported in the biomedical literature (21). In collaboration with the NIF (22, 23), OMICtools has already added unique RRIDs for each software tool and database.

During the development of OMICtools, we encountered two main problems. Firstly, it is often the case that basic program specifications (Table 1) are difficult to find or not supplied. We encourage developers to provide these details in their publications and on their Web sites. Secondly, Web sites containing deprecated tools are often simply closed without any indication. It would be useful to keep a web page indicating whether the program is temporarily or permanently unavailable. Such initiatives should be warmly acknowledged, as they promote the use of standards that could help the work of curators in the future.

**Table 1.** Examples of program specifications often unavailable that could help curation in the future

Program name	Bismark
Function	A tool to map bisulfite-converted sequence reads and determine cytosine methylation states
Initial contact	Felix Krueger
Created at	Babraham Bioinformatics
Access level	Public
Biological technology(ies)	Illumina platform
Operating system(s)	Linux, Mac OS X and Windows
Code maturity	Stable (for Bowtie and Bowtie2)
Language(s)	Perl
Requirement(s)	A functional version of Bowtie or Bowtie2 is required
Maintained?	Yes
Version	Version 0.12.2
Last updated	4 May 2014
Created	14 June 2010
Size	1.6 Mo
License	GNU GPL v3
Research article	(Krueger and Andrews, 2011) Bismark: a flexible aligner and methylation caller for Bisulfite-Seq applications. Bioinformatics.
PubMed URL	http://www.ncbi.nlm.nih.gov/pubmed/21493656

## Conclusions and future work

New research tools become available and existing tools are refined all the time. The aim of OMICtools is to provide the community with continuously updated information regarding bioinformatics tools, as no single algorithm can deal with all the ‘omic’ tasks that need to be accomplished. In addition, it is important to keep abreast of the continuous development of tools and avoid redundancy. Many common tasks and solutions often have been codified and made open-source. A 5-min OMICtools search often saves 2–3 days of implementing the codes from scratch. OMICtools can also help funding agencies to ensure that the submitted projects are high value-added.

OMICtools is an on-going project with many possibilities for interactions. Future plans include the addition of an advanced rating system, a tag search option, to convert the Web site to a responsive design and to create a free application for smartphones. Comments, questions and information about missing software are most welcome.
